# CD56 Is a Pathogen Recognition Receptor on Human Natural Killer Cells

**DOI:** 10.1038/s41598-017-06238-4

**Published:** 2017-07-21

**Authors:** Sabrina Ziegler, Esther Weiss, Anna-Lena Schmitt, Jan Schlegel, Anne Burgert, Ulrich Terpitz, Markus Sauer, Lorenzo Moretta, Simona Sivori, Ines Leonhardt, Oliver Kurzai, Hermann Einsele, Juergen Loeffler

**Affiliations:** 1 Department of Internal Medicine II, WÜ4i, University Hospital Wuerzburg, Wuerzburg, Germany; 2 Department of Biotechnology and Biophysics, Biocenter, Julius-Maximilian-University Wuerzburg, Wuerzburg, Germany; 3 Immunology Area, Pediatric Hospital Bambino Gesù, Rome, Italy; 4 Dipartimento di Medicina Sperimentale (DIMES) and Centro di Eccellenza per la Ricerca Biomedica, Universita‘ di Genova, Genova, Italy; 5 Septomics Research Centre, Friedrich Schiller University and Leibniz Institute for Natural Product Research and Infection Biology–Hans Knoell Institute, Jena, Germany

## Abstract

*Aspergillus* (*A*.) *fumigatus* is an opportunistic fungal mold inducing invasive aspergillosis (IA) in immunocompromised patients. Although antifungal activity of human natural killer (NK) cells was shown in previous studies, the underlying cellular mechanisms and pathogen recognition receptors (PRRs) are still unknown. Using flow cytometry we were able to show that the fluorescence positivity of the surface receptor CD56 significantly decreased upon fungal contact. To visualize the interaction site of NK cells and *A*. *fumigatus* we used SEM, CLSM and *d*STORM techniques, which clearly demonstrated that NK cells directly interact with *A*. *fumigatus* via CD56 and that CD56 is re-organized and accumulated at this interaction site time-dependently. The inhibition of the cytoskeleton showed that the receptor re-organization was an active process dependent on actin re-arrangements. Furthermore, we could show that CD56 plays a role in the fungus mediated NK cell activation, since blocking of CD56 surface receptor reduced fungal mediated NK cell activation and reduced cytokine secretion. These results confirmed the direct interaction of NK cells and *A*. *fumigatus*, leading to the conclusion that CD56 is a pathogen recognition receptor. These findings give new insights into the functional role of CD56 in the pathogen recognition during the innate immune response.

## Introduction

Invasive aspergillosis (IA), primarily caused by the mold *Aspergillus fumigatus*, is a devastating disease in immunocompromised patients suffering from hematological malignancies or undergoing allogeneic hematopoietic stem cell transplantation (HSCT)^1^. The mortality rate of HSCT patients diagnosed with IA ranges from 60–90%^2^ and the prognosis for long-term survival is extremely poor^3^. Recently, it was shown that HSCT patients with probable/proven IA had a delayed reconstitution of natural killer (NK) cells for more than a year post HSCT^4^. In addition, patients with severe IA were found to have a lower NK cell count compared to patients with well-controlled IA, suggesting that NK cells play a critical role in immunity to IA.

NK cells comprise 5–15% of the peripheral blood mononuclear cells (PBMCs) in healthy individuals and belong to the innate immune system^5^. Upon activation, NK cells release immune regulatory cytokines to stimulate other immune cells and display cytotoxicity directed against tumor or virus-infected cells by granule release^5^. NK cells are defined as CD56 positive and CD3 negative cells and can be distinguished into CD3^−^CD56^dim^CD16^+^ and CD3^−^CD56^bright^CD16^−^ cells. While CD56^dim^ cells are more cytotoxic, CD56^bright^ cells produce high levels of cytokines such as IFNγ and TNFα^6^. The function of NK cells is induced by the interplay of inhibitory and activating receptors^7^, leading to cytotoxicity directed against tumors and virus-infected cells. Besides the recognition of these cells, NK cells also recognize other infectious pathogens, become activated, and as a response induce either lysis of these pathogens or trigger activation of other immune cells by cytokine release^8–10^. Consequently, an important role of NK cells in the response to several fungal pathogens, including *A*. *fumigatus*, *Candida albicans*, *Cryptococcus neoformans* and *Mucorales* has been demonstrated^8, 11–16^.

Previous studies demonstrated that NK cells are activated by direct interaction with *A*. *fumigatus* germ tubes and hyphae^11, 14^. Upon activation NK cells release cytotoxic granules containing granzyme and perforin, which damage *A*. *fumigatus* hyphae^14^. Direct contact with *A*. *fumigatus* germ tubes induces IFNγ release of NK cells which interferes with fungal metabolic activity and growth^11^. Furthermore, studies in a neutropenic IA mouse model demonstrated that NK cell recruitment is essential for the clearance of the fungal infection and that IFNγ release by NK cells is critical for the immune defense during IA^17, 18^. Although NK cells have been shown to play a crucial role in host - pathogen interaction during *A*. *fumigatus* infection, the underlying mechanism and the NK cell recognition receptors have not been identified to date.

In this study, we examined the NK cell-*A*. *fumigatus* interaction to determine the PRR responsible for *A*. *fumigatus* recognition. None of the tested NK cell activating receptors demonstrated any changes in their expression levels on the cell surface when exposed to *A*. *fumigatus*. However, a significant reduction of CD56 fluorescence positivity of NK cells was observed upon contact with *A*. *fumigatus* germ tubes. Scanning electron microscopy (SEM), confocal laser scanning microscopy (CLSM) and *direct* stochastic optical reconstruction microscopy (*d*STORM)^19, 20^ were used to visualize the direct interaction of NK cells with *A*. *fumigatus* hyphae. We were able to demonstrate that CD56 was accumulating at the direct interaction site of NK cells with the fungus and that this re-organization of CD56 was dependent on the actin-cytoskeleton re-arrangement. Furthermore, we showed that blocking of CD56 reduced NK cell activation and partially restored CD56 fluorescence positivity of NK cells suggesting that CD56 is one recognition receptor for *A*. *fumigatus*.

## Results

### NK cell receptors are not altered in their expression while CD56 fluorescence positivity is significantly decreased upon fungal contact

To identify possible NK cell PRRs, the expression of several NK cell activating receptors and of the *A*. *fumigatus* PRRs TLR-2, TLR-4 and Dectin-1^21–23^ were analyzed in the presence of *A*. *fumigatus* after differenct incubation times using flow cytometry. Importantly, no difference in the expression of the mentioned receptors was noticed (Supplementary Fig. 1). Even so, NKp30 has been described as a PRR for fungal pathogens^8, 24^, no significant changes were detected in the presence of *A*. *fumigatus* (Supplementary Fig. 1).

**Figure 1:**
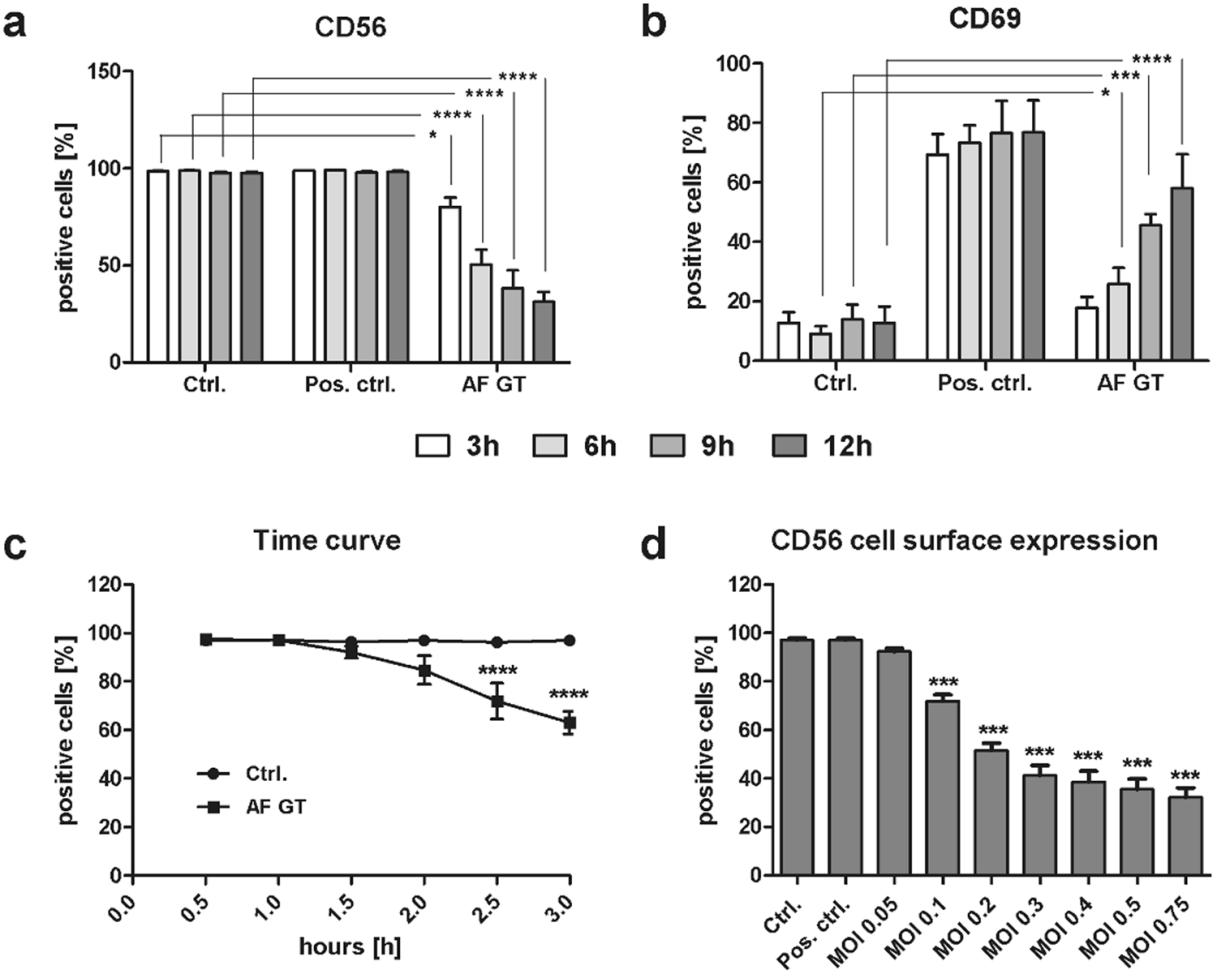
Reduction in CD56 positivity after fungal contact. NK cells were treated with 500 U/ml IL-15 and IL-2 (Pos. Ctrl.), with *A*. *fumigatus* germ tubes (AF GT, MOI 0.5) or left untreated (Ctrl.) for different periods of time. Flow cytometry was performed to analyze (**a**) CD56 expression (n = 4), (**b**) CD69 expression (n = 5), (**c**) time dependent down-regulation of CD56 (n = 5) and (**d**) CD56 expression after treatment with different MOIs of *A*. *fumigatus* germ tubes (AF GT, n = 3). NK cells were incubated for 3, 6, 9 and 12 h to determine (**a**) CD56 and (**b**) CD69 expression. To determine the time dependent down-regulation of CD56 NK cells were incubated for 0.5, 1, 1.5, 2, 2.5 and 3 h (**c**). To assess the effect of different fungal MOIs NK cells were incubated for 6 h (**d**). NK cells were defined as NKp46^+^CD3^−^. Data are represented as mean + SEM. Significant differences were are marked with an asterisk (*p < 0.05, ***p < 0.005, ****p < 0.001).

CD56 used in combination with CD3 is a well-known characterization marker to distinguish NK cells from other immune cells such as T-cells or monocytes^25, 26^. Surprisingly, we detected a prominent reduction of CD56 fluorescence positivity of NK cells after co-cultivation with *A*. *fumigatus* germ tubes compared to control NK cells (Fig. 1a and Supplementary Fig. 2). Additionally, NK cells were observed to upregulate the CD69 receptor (Fig. 1b) upon fungal contact, indicating NK cell activation^27^. Interestingly, reduction of CD56 fluorescence positivity of NK cells started as early as 2 h post incubation (Fig. 1c).

To evaluate whether this effect was dependent on the fungal MOI, we investigated the decrease of CD56 fluorescence positivity of NK cells at different MOIs 6 h after co-cultivation. A significant decrease of CD56 fluorescence positivity of NK cells (71.9%) was observed at a MOI of 0.1 compared to control NK cells (97%) (Fig. 1d).

**Figure 2:**
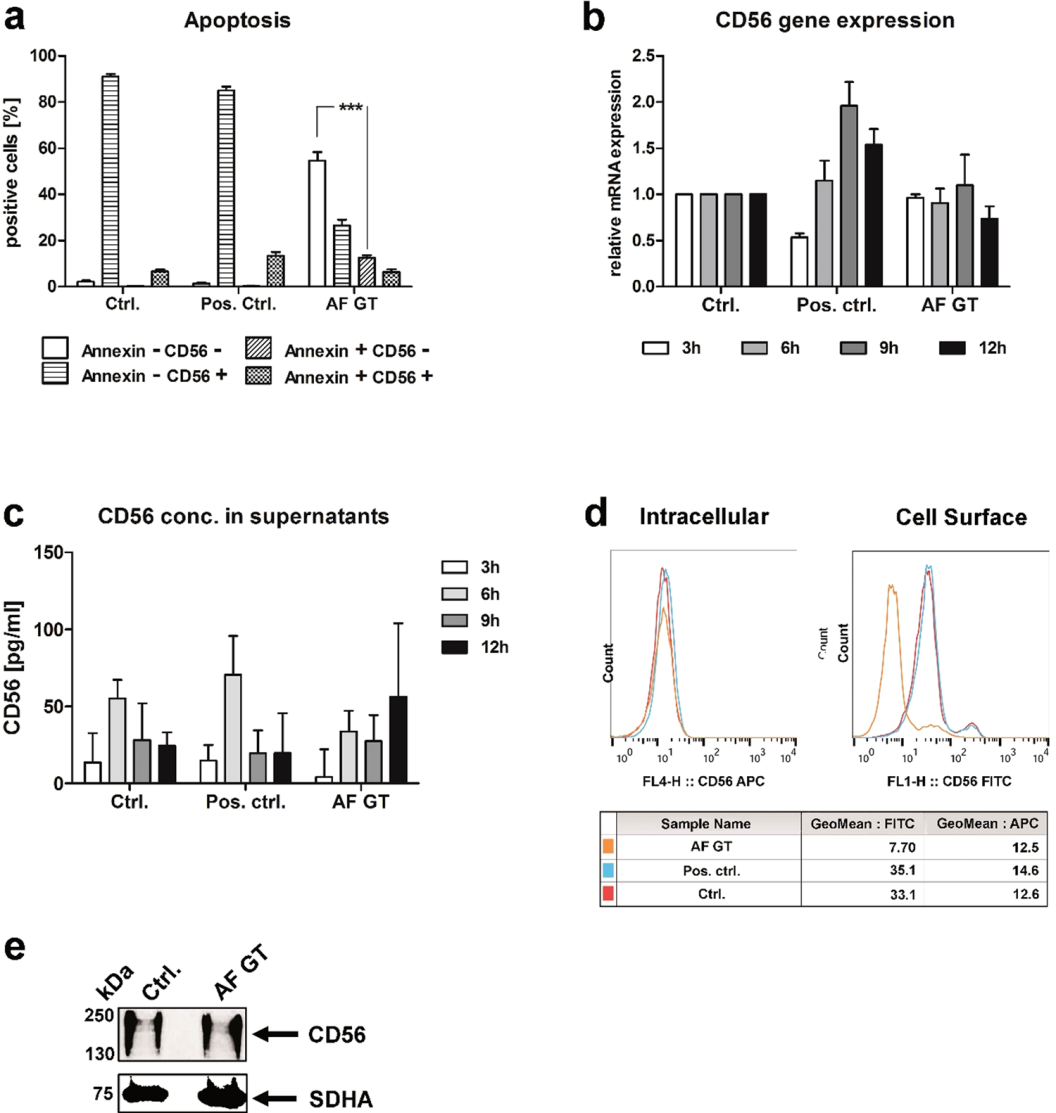
CD56 reduction is not induced by apoptosis, deregulation of protein and gene expression. NK cells were either treated with 500 U/ml IL-15 and IL-2 (Pos. ctrl.), with *A*. *fumigatus* germ tubes (AF GT, MOI 0.5) or left untreated (Ctrl.). (**a**) Induction of apoptosis was determined 9 h after co-cultivation and (**b**) mRNA/transcript expression after different incubation times was quantified by real-time RT-qPCR. (**c**) CD56 withdrawal was analysed by ELISA. (**d**) Intracellular and membranous CD56 expression was analysed by flow cytometry 6 h after co-cultivation, representative data of three independent experiments. Red line: NK cells (Ctrl.); blue line: NK cells treated with IL-15 and Proleukine (Pos. ctrl.); orange line: NK cells treated with *A*. *fumigatus* (AF GT). (**e**) Protein concentrations were visualized by western blot analyses 4 h after co-cultivation. Blots were cropped and image processing was performed by Adobe Photoshop software. Representative data of five independent experiments. Data of (**a**–**c**) are represented as mean + SEM for (**a**) n = , (**b**) n = and (**c**) n = 3). Significant differences are marked with an asterisk (***p < 0.005).

A potential mechanism that could provoke down-regulation of protein expression on the cell surface is apoptosis. Mycotoxins produced by *A*. *fumigatus* are not only able to inhibit DNA and RNA synthesis in affected cells, but can also induce apoptosis by cell membrane alterations^28^. To investigate whether the reduction of CD56 fluorescence positivity of NK cells was caused by the induction of apoptosis, NK cells were stained with Annexin V to identify apoptotic NK cells. NK cells confronted with *A*. *fumigatus* germ tubes for 9 h showed a reduction of CD56 fluorescence positivity (Fig. 2a), while only a few NK cells were both, CD56 negative and Annexin V positive. However, the CD56 negative NK cells were mostly negative for Annexin V (54.6%), indicating that apoptosis is not induced in these cells (Fig. 2a).

To better understand the mechanism of CD56 reduction, we determined CD56 gene expression in NK cells confronted with *A*. *fumigatus* germ tubes for different incubation times. In the control experiments the expression of CD56 mRNA was time-dependently increased after treatment with IL-15 and IL-2 (Fig. 2b) whereas the expression of CD56 mRNA in NK cells co-cultivated with *A*. *fumigatus* was not altered (Fig. 2b). These results indicated that gene expression was not differentially regulated by exposure to *A*. *fumigatus* and that a different mechanism is responsible for the reduction of CD56 positive NK cells.

To study potential CD56 shedding upon contact of NK cells with *A*. *fumigatus*, cell culture supernatants were collected from co-cultures after 3, 6, 9 and 12 h and the supernatants were tested for CD56 by ELISA. The level of CD56 in the supernatant was observed to be equal or below the smallest standard for all samples and no significant changes in CD56 levels were detected at any of the incubation times of the co-culture experiments (Fig. 2c). Thus, CD56 shedding from the cell surface during NK cell-*A*. *fumigatus* interaction could be excluded as a possible mechanism.

To investigate whether CD56 was internalized upon contact with *A*. *fumigatus*, NK cells were co-cultured with *A*. *fumigatus* germ tubes for 6 h before CD56 was surface- and intracellularly-labeled. CD56 on the cell surface of NK cells cultured with germ tubes was significantly reduced compared to control NK cells while the intracellular CD56 signal did not change (Fig. 2d). Even when intracellular CD56 signal was measured after preceding trypsination of surface receptors, CD56 levels did not change (Supplementary Fig. 4). The protein concentration of CD56 was evaluated to investigate whether CD56 protein was degraded or protein synthesis was inhibited in NK cells exposed to *A*. *fumigatus* potentially due to the release of mycotoxins^28^. Cells were harvested after co-culture with *A*. *fumigatus* for 4 h, and protein lysates were subsequently prepared for western blot analysis. The signal of the CD56 protein in *A*. *fumigatus* treated NK cells was comparable to the one of control NK cells (Fig. 2e, Supplementary Fig. 3). Since we detected a decrease in CD56 fluorescence positivity of NK cells but not by western blotting, we concluded that this effect might be the consequence of fewer antigen-antibody interactions due to sterical problems.

### NK cells directly interact with live *A*. *fumigatus* and CD56 is re-organized to the interaction site via actin filaments

NK cells were shown to release IFNγ and perforin upon direct contact with *A*. *fumigatus* ^11, 14^, thus we investigated the role of direct contact on the reduction of CD56 fluorescence positivity of NK cells.

**Figure 3:**
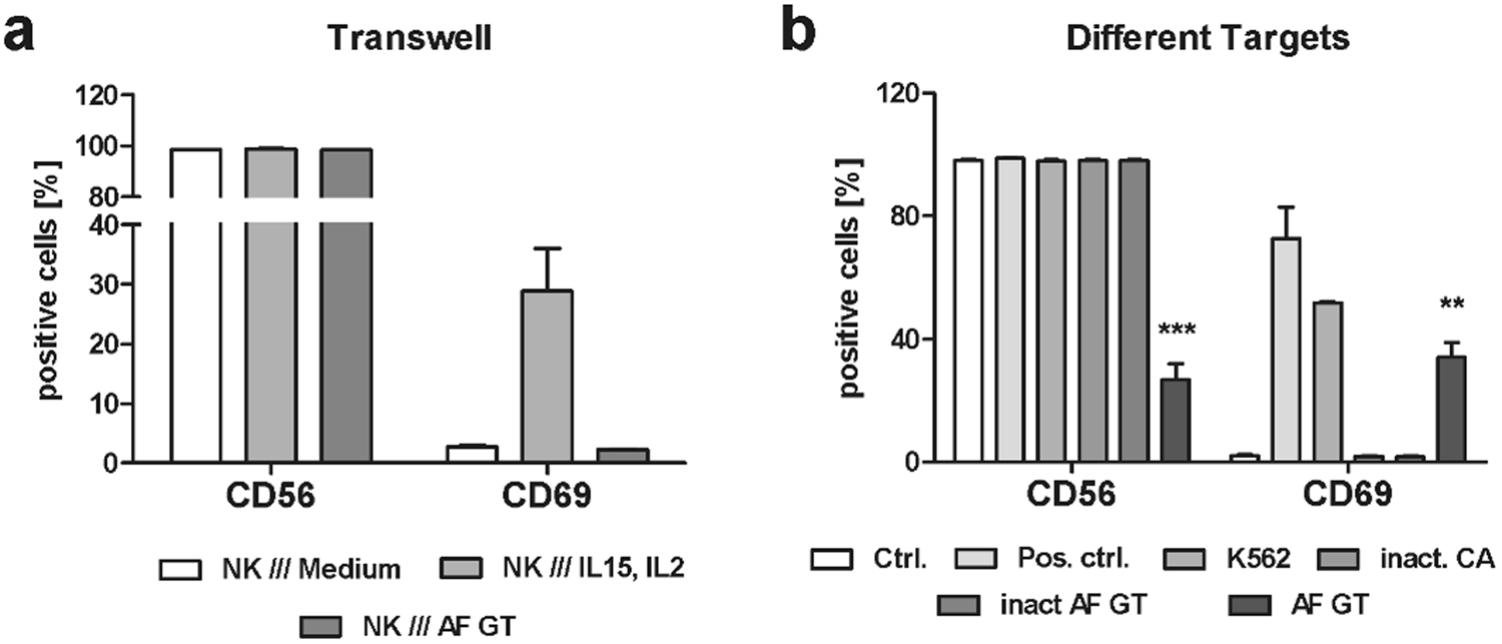
Reduction of CD56 is a result of the direct fungal contact. (**a**) NK cells were separated from fungal germ tubes (AF GT, MOI 0.5), medium supplemented with 500 U/ml IL-15 and IL-2 (Pos. ctrl) or medium (Ctrl.) by a transwell membrane (n = 3). (**b**) NK cells were cultured alone (Ctrl.), with 500 U/ml IL-15 and IL-2 (Pos. ctrl.), with K562 at an effector to target ratio of 5:1 (K562), with inactivated *C*. *albicans* (inact. CA, MOI 0.5) and with live and inactivated *A*. *fumigatus* germ tubes (AF GT, inact. AF GT, MOI 0.5, n = 6). Data are represented as mean ± SEM. Significant differences are marked with an asterisk (**p < 0.01, ***p < 0.005).

Co-cultures were prepared, separating NK cells and *A*. *fumigatus* germ tubes with transwell permeable membranes. The membranes were small enough to prohibit the contact of cells and *A*. *fumigatus* but large enough for molecules such as cytokines or mycotoxins to diffuse into the lower compartment. Expression of CD56 and CD69 on the surface of NK cells was determined after a 6 h cultivation. As a positive control, IL-15 and IL-2 were added to the transmembrane system to activate NK cells (Fig. 3a). Separation of *A*. *fumigatus* germ tubes and NK cells by the transwell membrane did not induce NK cell activation, nor was CD56 fluorescence positivity of NK cells reduced (Fig. 3a). These results demonstrate that the reduction of CD56 fluorescence positivity of NK cells was not mediated via a fungal-derived soluble factor but was depended on direct contact with *A*. *fumigatus*.

To determine whether the decrease of CD56 fluorescence positivity of NK cells was regulated by the interaction with live germ tubes, NK cells were co-cultivated with inactivated *A*. *fumigatus* germ tubes, inactivated *C*. *albicans*, and live *A*. *fumigatus* germ tubes. NK cells exhibit cytotoxicity against tumor cells and become activated upon contact with these cells^29^, therefore NK cells cultivated in the presence of the cancer cell line K562 served as a positive control. Cells were incubated for 12 h with the different targets. Then, CD56 and CD69 fluorescence positive cells were determined. K562 cells induced a significant activation of NK cells that was comparable to the activation of NK cells treated with IL-15 and IL-2, but showed no decrease of CD56 (Fig. 3b). Inactivated *C*. *albicans* and *A*. *fumigatus* did not induce the reduction of CD56 fluorescence positivity nor activate NK cells (Fig. 3b). Live *A*. *fumigatus* germ tubes activated NK cells and significantly reduced the number of CD56 fluorescence positive NK cells (Fig. 3b), suggesting that the decrease of CD56 fluorescence positivity of NK cells was only induced by live *A*. *fumigatus*.

From these experiments we hypothesized that CD56 interacts as a recognition receptor with *A*. *fumigatus*.

**Figure 4:**
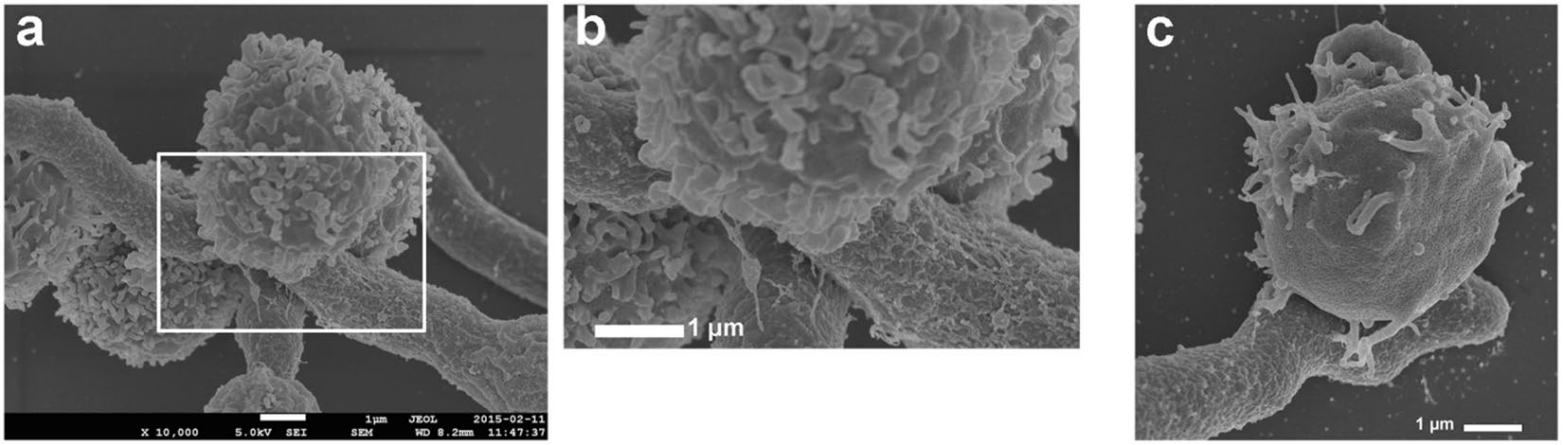
Direct contact with *A. fumigatus* hyphae. NK cells-*A*. *fumigatus* 3 h co-culture specimens were inspected with a field emission SEM using a detector for secondary electrons at 5 kV and a magnification of ×10,000. Representative result of two independent experiments performed in duplicates. Picture (**b**) is a zoom in of (**a**), illustrating the interaction site of NK cells with *A*. *fumigatus* hyphae. Scale bar for (**a**–**c**): 1 µm.

A further potential mechanism hypothesized for the reduction of CD56 fluorescence positivity of NK cells upon contact with *A*. *fumigatus* germ tubes is that CD56 acts as an interaction receptor for *A*. *fumigatus*. SEM, CLSM and *d*STORM microscopy were used to determine whether or not human NK cells interact directly with *A*. *fumigatus* hyphae and if CD56 is re-located during this interaction. NK cells were cultured alone or in the presence of *A*. *fumigatus* germ tubes for 3 h and SEM pictures were taken from NK-*A*. *fumigatus* co-cultures. In fact, NK cells were observed interacting directly with *A*. *fumigatus*, and the interaction site was mostly at the hyphal part of the fungus (Fig. 4). We observed a close interaction of NK cells with *A*. *fumigatus* suggesting that NK cells recognize the fungus via specific receptors.

**Figure 5:**
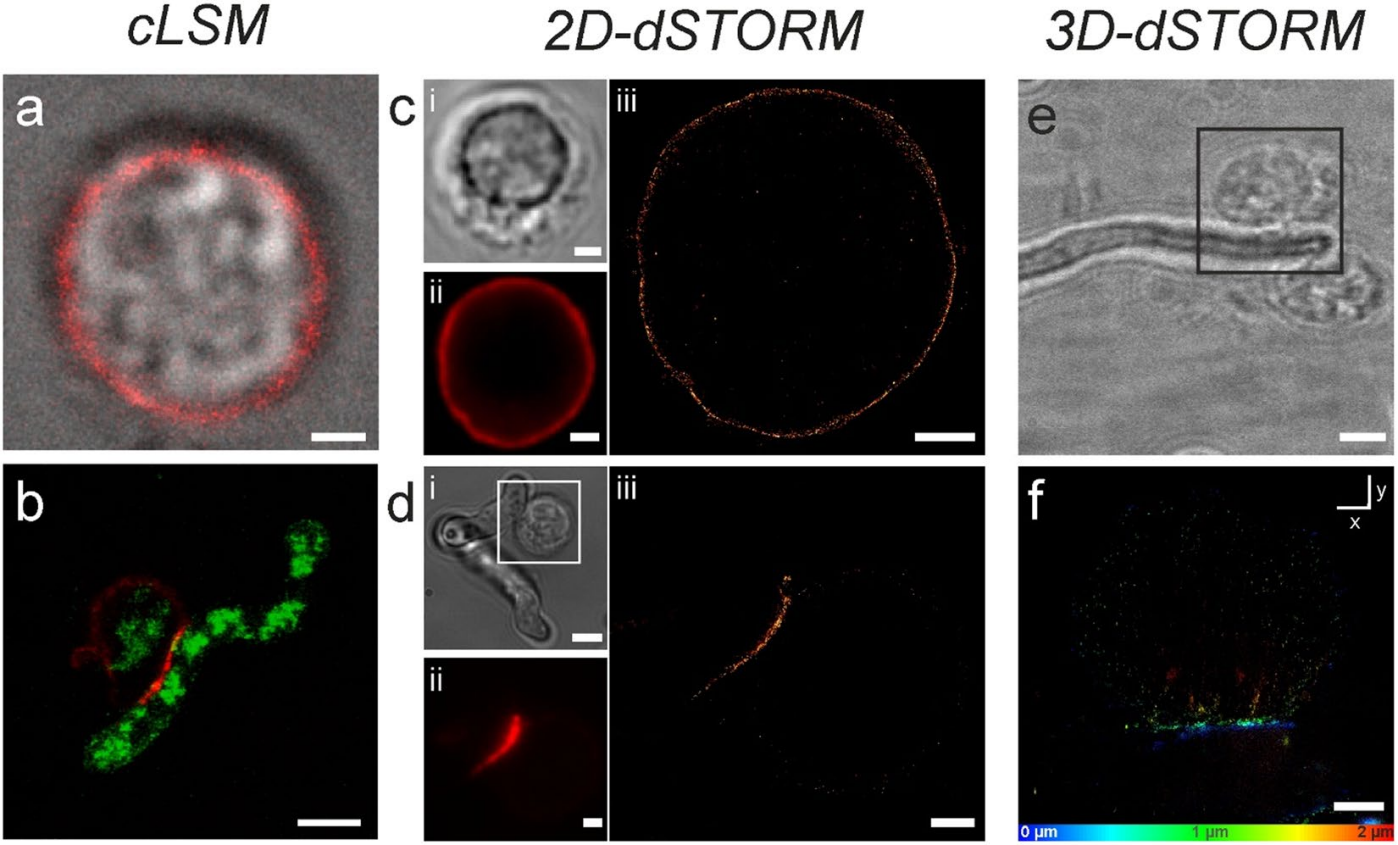
CD56 re-organization to the interaction site. NK cells and NK cells-*A*. *fumigatus* co-cultures were visualized with (**a**,**b**) CLSM, (**c**,**d**) 2D *d*STORM, and (**e**,**f**) 3D *d*STORM after staining with anti-CD56. NK cells were either left untreated (**a**,**c**) or incubated with *A*. *fumigatus* germ tubes (MOI 0.5) for 3 h (**b**,**d**–**f**). (**b**) Auto fluorescence was recorded to visualize NK cell-*A*. *fumigatus* interaction. Transmitted light pictures (ci, di), (**e**), widefield fluorescence images (cii, dii), and *d*STORM images (ciii, diii) of the interaction site were compared. In (**f**) the x-y projection of a reconstructed 3D dSTORM image is given which is color coded in z. (**e**) The transmitted light picture is a zoom out of (**f**) illustrating two NK cells which interact with *A*. *fumigatus*. Scale bars: 1.5 μm (**c**, dii,iii), 2 μm (**b**,**f**), 3 μm (**a**,**e**), 4 μm (di), and 5 μm (**b**). 3D stack showing interaction site of NK cell *and A*. *fumigatus* (**e**,**f**). NK cells were incubated for 3 h with *A*. *fumigatus*, thereafter stained with anti CD56-Alexa Fluor 647, and visualized in 3D.

**Figure 6:**
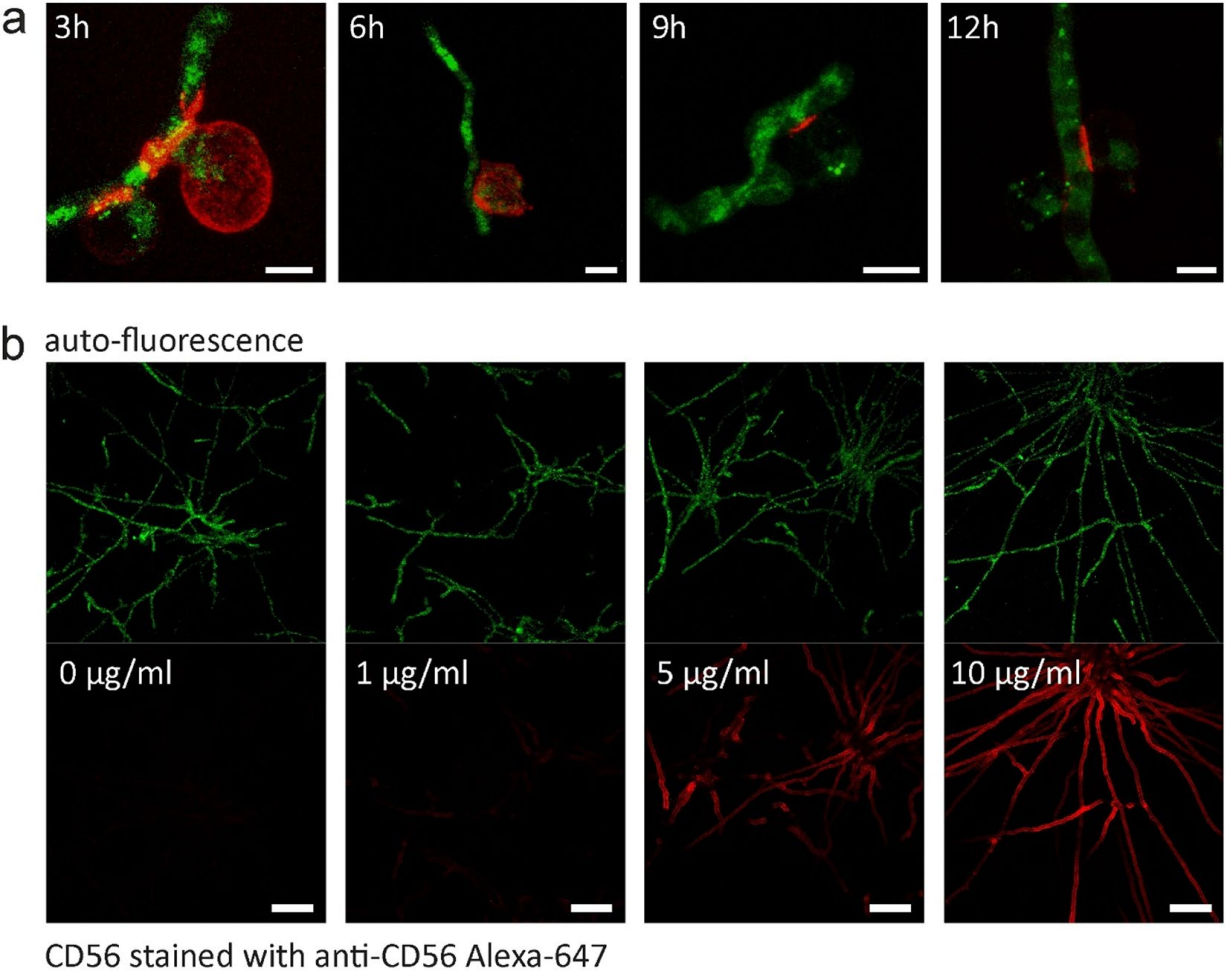
Time course of the NK cell*-A. fumigatus* interaction and direct binding of CD56 to the fungus. (**a**) NK cells were cultured with *A*. *fumigatus germ tubes* (MOI 0.5) for 3, 6, 9 and 12 h. CLSM pictures were taken from these co-cultures. (**b**) Fungal hyphae were incubated for 6 h with 0, 1, 5, and 10 μg/ml soluble CD56 and were afterwards stained with anti-CD56 antibody. Alexa 647-labeled anti-CD56 antibody was used to visualize the distribution of CD56, while germ tubes could be detected via their auto fluorescence. Scale bars represent 5 μm (**a**) and 30 µm (**b**).

To further confirm this observation and the possibility that CD56 is a recognition receptor we performed CLSM and super-resolution *d*STORM microscopy. After the cultivation of NK cells in the presence of *A*. *fumigatus* for different incubation times, CD56 localization was determined. Indeed, NK cells incubated with *A*. *fumigatus* revealed a strong CD56 signal at the contact site whereas other parts of the plasma membrane exhibited only a weak signal (Fig. 5b,d–f). In contrast, the CD56 fluorescence signal in control NK cells was homogenously distributed on the plasma membrane (Fig. 5a,c). 3D-*d*STORM analysis revealed a concentration of CD56 fluorescence at the interaction site and in lanes surrounding the interaction site (Fig. 5e,f; Video 1). We further observed that CD56 relocalization occurs in a time dependent manner (Fig. 6a). At 3 h and 6 h after initiation of co-culture, CD56 signal is observed at the fungal interface and still ubiquitously distributed in the remaining NK cell membrane which is not interacting with the fungus. At 9 h and 12 h, the CD56 signal is detected at the fungal interface but not in the remaining NK cell membrane anymore (Fig. 6a). To proof the result that the interaction site is increasing time dependently, we measured the length and amount of CD56 stained interaction sites after 3, 6, 9, and 12 h of co-culture, respectively. Indeed, the highest counts and the greatest lengths of interaction sites were detectable after 12 h of co-cultivation (Supplementary Fig. 5), confirming that CD56 is re-localized to the fungal interface in a time dependent manner. To elucidate whether CD56 is directly interacting with the fungus or if CD56 accumulation at the fungal interface is mediated by indirect mechanisms, we co-cultured live *A*. *fumigatus* germ tubes with different concentrations of soluble CD56 protein for 6 h and afterwards stained the samples with a fluorescent anti-CD56 antibody (Fig. 6b). As a control, *A*. *fumigatus* germ tubes were cultured alone and were stained with fluorescent anti-CD56 antibody to exclude the possibility of unspecific antibody binding to the fungus (Fig. 6b). In contrast to the negative control, incubation of *A*. *fumigatus* with soluble CD56 resulted in a staining of fungal structures after incubation with anti-CD56 antibody (Fig. 6b). These experiments showed that CD56 is time-dependently relocalized to the fungal interface and directly binds the fungus.

**Figure 7:**
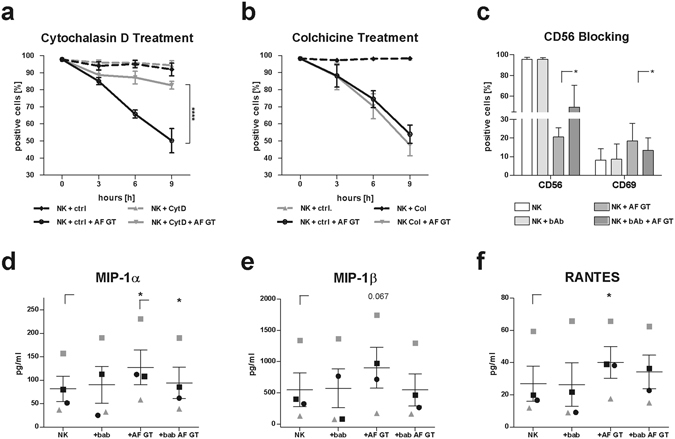
CD56 mediated fungal recognition is dependent on actin and CD56 blocking inhibits NK cell function. NK cells were incubated with (**a**) cytochalasin D (Cyt. D, n = 4), (**b**) colchicine (Col., n = 3) and (**c**) CD56 blocking antibody (bAb, n = 4) prior to co-cultivation with the fungus (AF GT). Percentage of CD56 positive cells was determined after an incubation with the fungus for 0, 3, 6, and 9 h. A paired student’s t-test was performed to compare NK + ctrl + AF GT against NK + CytD + AF GT or NK + Col + AF GT (**a**,**b**). Control samples were cultured in the presence of the corresponding control solution. Percentage of CD56 and CD69 positive cells was assessed after a co-cultivation for 9 h. CD56 and CD69 expression was analysed using flow cytometry. NK cells were defined as NKp46^+^CD3^−^. Supernatants derived from CD56 blocking experiments were analysed by ProcartaPlex^TM^ multiplex immunoassays (n = 4, **d**–**f**). The concentration of (**d**) MIP-1α, (**e**) MIP-1β and (**f**) RANTES detectable in supernatants is displayed in pg/ml. A paired student’s t-test was performed to compare (**c**) NK + AF GT against NK + bAb + AF GT and (**d**–**f**) NK against + AF GT and + AF GT against + bAb AF GT. Data are represented as mean ± SEM. Significant differences are indicated by an asterisk (*p < 0.05, ****p < 0.001).

It is well known that NK cells encounter the cytoskeleton when they recognize and lyse target cells^30^. While the actin cytoskeleton plays a role in the early recognition of target cells and enables receptor reorganization^30^, cell lysis occurs to later time points and is mediated by the transport of lytic granules to the target interface via microtubules^30^. To investigate whether the actin or the microtubule cytoskeleton plays a role in the re-organization of CD56, we treated NK cells with either actin or microtubules inhibiting agents and then co-cultured NK cells with *A*. *fumigatus* germ tubes for 0, 3, 6, and 9 h, respectively. Cytochalasin D is preventing actin polymerization and elongation by binding to existing actin filaments^31^, whereas colchicine binds to soluble tubulin dimers and thereby inhibits microtubule polymerization^32^ and thus prevents the transport of granules to the membrane^33^. NK cells treated with cytochalasin D compared to control NK cells did not show any differences in the CD56 fluorescence positivity of NK cells (Fig. 7a). However, when NK cells were challenged with the fungus we detected significantly less CD56 reduction of fluorescence positivity in cytochalasin D treated samples compared to controls, concluding that relocalization of CD56 is inhibited (Fig. 7a).

In contrast, treatment with colchicine did not result in any increase of the CD56 fluorescence positivity of NK cells in the presence of fungus compared to untreated NK cells (Fig. 7b).

Consequently, inhibition of actin polymerization but not inhibition of the microtubules impaired CD56 re-localization to the fungal interaction site concluding that CD56 plays a role in the early fungal recognition.

To further functionally analyze the role of CD56, we blocked CD56 on the NK cell surface using an anti-CD56 blocking antibody before co-cultivation of NK cells with *A*. *fumigatus* germ tubes for 9 h (Fig. 7c). The blocked CD56 receptor was still recognized by the flow cytometric antibody directed against CD56, since NK cells treated with the blocking antibody displayed the same percentage of CD56 positivity as unblocked NK cells (Fig. 7c). In the presence of the fungus, CD56 blocking restored the number of CD56 fluorescence positive NK cells to 55% after fungal co-culture compared to 20% when CD56 was not blocked (Fig. 7c). Interestingly, treatment with CD56 blocking antibody significantly decreased the fungal-induced activation of NK cells (13.3%) compared to NK cells on which CD56 was not blocked (18.5%) (Fig. 7c).

To further characterize the effects on CD56 blocking in the NK cell response to the fungus, culture supernatants from CD56 blocked and unblocked NK cells were analyzed by multiplex immunoassay (Fig. 7d–f). Challenging unblocked NK cells with *A*. *fumigatus* provoked a significant induction in the secretion of macrophage inflammatory protein (MIP)-1α (CCL3) and RANTES (CCL5) while MIP-1β showed a tendency (p = 0.067) to be higher secreted after fungal stimulation (Fig. 7d–f). This fungal mediated cytokine secretion was reduced when CD56 was blocked on the NK cell surface compared to the unblocked NK cells in the presence of the fungus. We detected no significant differences between CD56 blocked NK cells with and without fungal stimulation (Fig. 7d–f). Indeed, we further observed a significant reduction for MIP-1α secretion in CD56 blocked NK cells challenged with the fungus compared to unblocked NK cells in the presence of the fungus (Fig. 7d). These blocking experiments verified the functional role of CD56 confirming that CD56 is a recognition receptor for *A*. *fumigatus*.

### Reduction of CD56 fluorescence positivity is detectable in the presence of other *Aspergillus* species

**Figure 8:**
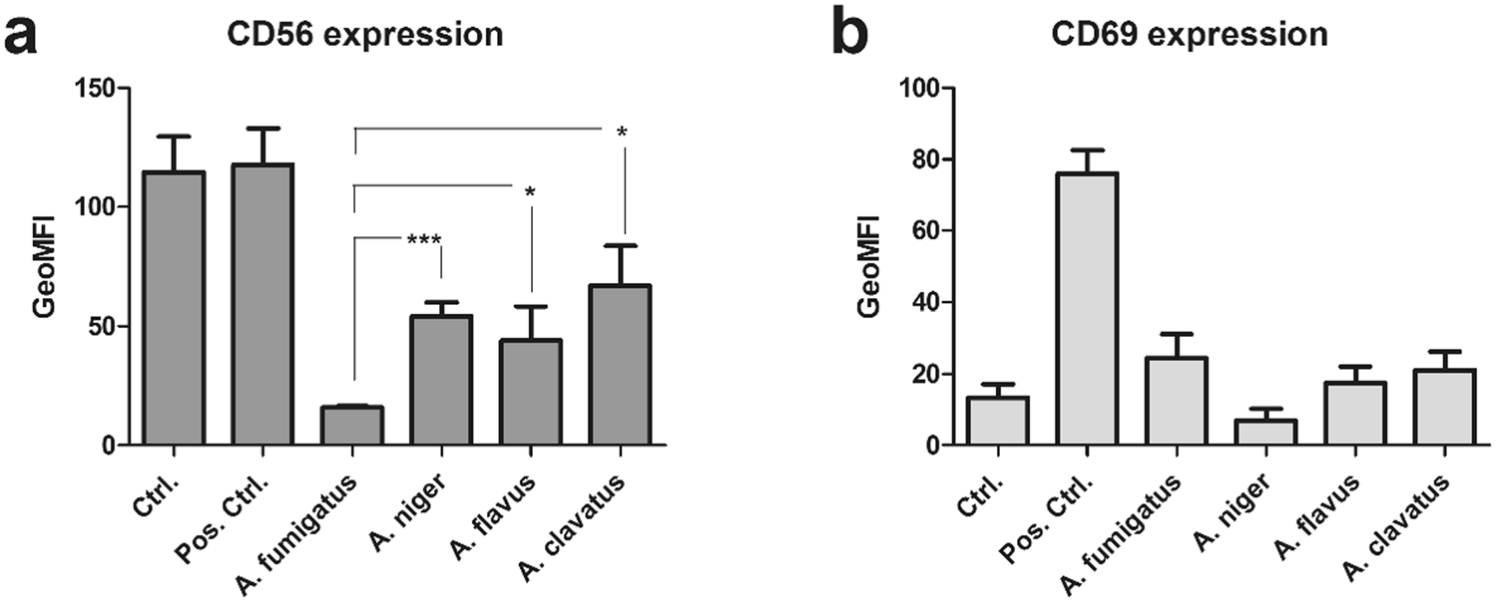
The reduction of CD56 is not as stringent for other Aspergillus species as it is for *A. fumigatus*. Primary NK cells were pre-treated overnight with IL-2 (1000 U/ml). Pre-treated NK cells were cultured in the presence of germ tubes of *A*. *fumigatus*, *A*. *niger*, *A*. *flavus* or *A*. *clavatus* at MOI 0.5, or were left untreated (Ctrl.) for 6 h. NK cells treated with 500 U/ml IL-15 and IL-2 served as control for NK cell activation (Pos. ctrl.). The expression of CD56 (**a**) and CD69 (**b**) was analysed using flow cytometry. NK cells were defined as NKp46^+^CD3^−^. Data are represented as mean of the geometric mean fluorescence intensity (GeoMFI) + SEM of n = 4 independent experiments. Significant differences between AF GT treated NK cells and NK cells treated with the other *Aspergilli* are indicated by an asterisk (*p < 0.05; **p < 0.01; ***p < 0.005; ****p < 0.001).

To investigate whether CD56 fluorescence positivity of NK cells was also reduced in the presence of other *Aspergillus* species, pre-stimulated NK cells were cultured with *A*. *niger*, *A*. *clavatus*, *A*. *flavus* and *A*. *fumigatus* germ tubes for 6 h. Expression of CD56 and CD69 was then determined using flow cytometry. All *Aspergillus* species tested induced a reduction of CD56 (Fig. 8a). However, NK cells confronted with *A*. *fumigatus* germ tubes displayed a significantly higher decrease of CD56 fluorescence positivity of NK cells compared to *A*. *niger*, *A*. *flavus* and *A*. *clavatus* (Fig. 8a). NK cells co-cultivated with *A*. *fumigatus*, *A*. *flavus* and *A*. *clavatus* displayed an increase in the CD69 expression indicating NK cell activation whereas NK cells treated with *A*. *niger* showed a decrease in CD69 expression compared to control NK cells (Fig. 8b). These results suggest that *Aspergillus* species express a specific molecule on their surface recognized by CD56 on NK cells.

## Discussion

This study is the first to visualize the direct interaction of NK cells and *A*. *fumigatus* and to show that CD56 has a functional role during fungal recognition. NK cells can recognize fungal pathogens and induce their lysis^8–10, 12^. Besides their antifungal activity towards *C*. *albicans*, *C*. *neoformans*, *Paracoccidioides brasiliensis* ^34^ and *Coccidioides immitis* ^35^, it was shown that NK cells recognize *A*. *fumigatus* and display antifungal activity directed against the hyphae^11, 14^. However, the mechanism of this interaction is still poorly understood.

Previous studies demonstrated that NK cells form direct conjugates with *C*. *neoformans* ^35, 36^ and that NKp30 and NKp46 act as fungal PRR^8, 24^. These publications and our previous studies^11^ suggested that the interaction of NK cells and *A*. *fumigatus* is mediated by a PRR. Unexpectedly, neither experimentally tested NK cell activating receptors nor the known fungal recognizing receptor NKp30 were modulated upon co-culture with *A*. *fumigatus*. Surprisingly, we observed a striking decrease of CD56 fluorescence positivity on the NK cell surface upon exposure to the fungus. By quantifying the NCAM/CD56 protein concentration in the supernatant of NK cells-*A*. *fumigatus* co-cultures, we could exclude that NCAM/CD56 was neither shed from the cell surface nor was the secreted isoform of NCAM/CD56 expressed.

Mycotoxins have an impact on the mRNA and protein expression of host cells but our analyses clearly showed that the secreted mycotoxins have no influence on the expression level of CD56 on transcriptome and protein level of NK cells.

On neuronal cells, NCAM/CD56 can be endocytosed and is then mostly recycled to the cell surface whereas a minority of the endocytosed NCAM/CD56 is degraded^37^. Analyses showed that NCAM/CD56 was not internalized upon contact with *A*. *fumigatus*. Thus, we speculated about a potential binding of CD56 to the fungus that is masking the molecule as it was seen as well for NKp30 in the studies of Li *et al*.^8^. Indeed, we were able to show a direct interaction of NK cells with live *A*. *fumigatus* by SEM, CLSM and super-resolution *d*STORM microscopy, which showed that NCAM/CD56 distribution on the NK cell surface markedly changed after fungal contact. In addition, NCAM/CD56 re-location was observed until the complete CD56 signal was detected at the fungal interaction site and the lengths and amounts of the interaction site increased over time (Supplemetary Fig. 4). Furthermore, we could exclude that CD56 is shed from the NK cell surface and bound to *A*. *fumigatus* by CLSM microscopy. In NK cells-*A*. *fumigatus* co-cultures we were not able to detect any CD56 outside of the interaction site with NK cells. NCAM/CD56 positive NK cells were also decreased in the presence of other *Aspergillus* species but less when compared to *A*. *fumigatus* suggesting that *A*. *fumigatus* is expressing a CD56 ligand with a higher abundance.

CD56 was identified as the 140 kDa isoform of the human neural-cell adhesion molecule (NCAM)^25^. Two isoforms (140 kD and 180 kD) of NCAM show transmembrane binding and have intracellular domains while the 120 kD isoform has a glycosyl-phosphatidylinositol membrane anchor but no intracellular domains^38^. While the three isoforms have different C-termini, the N-terminal extracellular domains are identical in all three isoforms^39^. Therefore, we used the 120 kD isoform to test whether CD56 is directly interacting with *A*. *fumigatus*. By microscopy, we showed that soluble CD56 directly binds in a concentration dependent manner to growing *A*. *fumigatus* structures, confirming our previous observations and hypothesis.

Blocking of CD56 did not only reduce fungal mediated NK cell activation but further inhibited the amount of secreted cytokines. Chemokines like MIP-1α (CCL3), MIP-1β (CCL4), and RANTES (CCL5) are secreted by human blood NK cells^40, 41^. CCL3, 4, and 5 modulate the migratory behavior of leukocytes and their importance in cryptococcal infections was highlighted by the study from Huffnagle and McNeil (1999)^42^. Huffnagle and McNeil showed that depletion of either MIP-1α or the common MIP-1α, MIP-1β and RANTES receptor CCR5 conferred to a higher fungal burden and inhibited leukocyte recruitment in the central nervous system of knockout mice^42^. The role of the CCR5 ligands CCL3, 4, and 5 was also highlighted in another study that reported an abolished NK cell accumulation at sites of infection in CCR5^−/−^ mice^43^. Detection of fewer levels of MIP-1α, MIP-1β, and RANTES in supernatants derived from samples in which NK cells were blocked with CD56 and challenged with the fungus compared to co-cultures in which CD56 was not blocked is suggesting a crucial role for these chemokines and NK cells in the immune response directed against the fungus.

Based on these results and the previous publications we conclude that CD56 is involved in the secretion of CCL3, 4, and 5 to recruit further leukocytes such as NK cells, monocytes and neutrophils to sites of *A*. *fumigatus* infections.

Recently, Mace *et al*. demonstrated that CD56 is accumulated at the developmental synapse which is formed at stromal cells and that CD56 is co-localized with F-actin^44^. Showing that CD56 re-localization is dependent on the actin cytoskeleton, we could confirm the findings of Mace *et al*.^44^ leading to the suggestion that CD56 plays a crucial role in the recognition of pathogens.

A recent study published by Voigt *et al*. demonstrated a decrease of NCAM/CD56 expression in the presence of live *C*. *albicans* ^16^. In addition, it was shown that the surface protein gp63 of *Leishmania* further reduces CD56 fluorescence positivity, indicating that NCAM/CD56 plays a functional role in the recognition of eukaryotic and prokaryotic pathogens expressing a specific molecule on their cell surface. These publications further strengthen our hypothesis that NCAM/CD56 is a pathogen recognition receptor^45^.

The functional role of NCAM/CD56 expressed by NK cells referring to NK cell cytotoxicity against tumor cells has been controversially discussed. It was observed that NCAM/CD56 had no impact on the lysis effect of target cells^46, 47^ whereas on the other hand, other reports demonstrated that the cytotoxicity of NK cells interacting with NCAM-expressing target cells is enhanced by NCAM/CD56^48, 49^. These reports suggested a functional role of NCAM/CD56 in the recognition of target cells and in the induction of cytotoxicity. These observations and our findings suggest that NCAM/CD56 is a pathogen recognition receptor and plays a functional role for the NK cell cytotoxicity in the innate immune response. Our study provides novel insights in the interaction of NK cells and *A*. *fumigatus* as well as in NK cell biology.

## Methods

### Cell culture

Human PBMCs were isolated from fresh blood of healthy volunteers giving written consent by a Ficoll standard density gradient centrifugation (Biochrom AG). Usage of the human blood specimens was approved by the Ethical Committee of the University Hospital Wuerzburg. NK cells were isolated from PBMCs using MACS NK negative selection kit (Miltenyi Biotec) and were cultured in RPMI 1640 (Invitrogen) supplemented with 10% heat-inactivated fetal bovine serum (FBS) and 120 µg/ml gentamicin (Refobacin; Merck) at 37 °C and 5% CO_2_. K562 cells were cultured under the same conditions as human NK cells. NK cells were stimulated overnight with 1000 U/ml recombinant human (rh) IL-2 (Proleukin, Novartis).

### Infection conditions

*A*. *fumigatus* (ATCC 46645), *A*. *flavus* (CBS 625.66) and *A*. *clavatus* (CBS 114.48) conidia and germ tubes were prepared as described previously^50^. *A*. *niger* (CBS 553.65) germ tubes were prepared in RPMI with 10% FCS overnight. NK cells were incubated with live *A*. *fumigatus* germ tubes at a multiplicity of infection (MOI) of 0.5 at 37 °C for 3, 6, 9 or 12 h. For one experimental setup NK cells were incubated with *A*. *fumigatus* germ tubes and *C*. *albicans* (Wildtype SC5314) hyphae that were inactivated as previously described^51^.

### Flow Cytometry

The purity of isolated NK cells (>95%) and expression of surface receptors were determined by flow cytometry using a FACSCalibur (BD-Biosciences). NK cell population was defined as NKp46^+^CD3^−^ cells.

Changes in the expression of NK cell receptors were examined using the following antibodies: anti-NKp30 PE (Biolegend), anti-NKp44 PE (Biolegend), anti-NKG2D PE (BD-Biosciences), anti-CD16 FITC (Miltenyi Biotec), anti-CD56 FITC and APC (BD-Biosciences), anti-2B4 FITC (Biolegend), anti-NTB-A PE (Biolegend), anti-Dectin-1 PE, anti-TLR-2 PE and anti-TLR-4 PE (BD-Biosciences). Apoptotic NK cells were assessed after an incubation time of 9 h using Annexin V FITC (BD-Biosciences) after staining cells with the surface marker antibodies anti-NKp46 PE, anti-CD3 PerCP and anti-CD56 APC. Intracellular expression of CD56 after incubation with germ tubes for 6 h was investigated by firstly staining CD56 on the cell surface with anti-CD56 FITC (BD-Biosciences). Then, cells were fixed (4% formaldehyde), permeabilized (Wash Perm, BD-Biosciences) and intracellular CD56 was stained using anti-CD56 APC (BD-Biosciences). To remove surface markers, NK cells were incubated in 0.5% trypsin-EDTA (Sigma) for 30 min at 37 °C before samples were stained. All data were analyzed with FlowJo software (Tree Star Inc.).

### Western Blot Analysis

NK cells were incubated for 4 h either alone or with *A*. *fumigatus* germ tubes at MOI 0.5 under standard culture conditions, before a total crude protein extraction was performed^52^. Proteins were separated on 12% SDS-PAGE gels, blotted onto nitrocellulose membrane and CD56 was detected using an anti-CD56 antibody (Cell Signaling, clone 123C3). Succinate dehydrogenase complex, subunit A (SDHA) served as a loading control (Cell Signaling).

### Enzyme Linked Immunosorbent Assay (ELISA) and multiplex immunoassay

To quantify the CD56 concentration in co-culture supernatants, an ELISA assay was performed according to the manufacturer’s instructions (Abcam). Secretion of cytokines and other proteins released by NK cells were assessed in cell-free supernatants using the ProcartaPlex^TM^ multiplex immunoassay from Affymetrix eBioscience. The analyses were performed according to the manufacturer’s instructions using the Bio-Plex 200 System from Bio-Rad. CCL 3 ELISA (MIP-1α, R&D), CCL 4 ELISA (MIP-1β, R&D), and CCL 5 ELISA (RANTES, Biolegend) were performed as described in the manufacture’s manual. The ELISA was performed according to the manufacture’s manual using the NanoQuant (infinite M200 Pro, Tecan).

### Transwell experiments

600 µl RPMI containing 0.6 × 10^6^ NK cells were seeded in 24-well plates, then a transwell membrane insert (Corning) with a pore size of 0.4 µm was inserted into each well and 100 µl RPMI were added per transwell. Depending on the condition, the 100 µl RPMI were either pure, supplemented with 500 U/ml IL-15 and IL-2, or enriched with *A*. *fumigatus* germ tubes MOI 0.5. After 6 h, NK cells were harvested and receptor expression of CD56 and CD69 was monitored by flow cytometry.

### CD56 blocking

A GPR165 (IgG2a) monoclonal blocking antibody was kindly provided by Daniela Pende and Alessandro Moretta and CD56 was blocked as previously described^53^. NK cells (4 × 10^6^ cells/ml) were incubated in CD56 blocking antibody (10.9 μg/ml) or IgG2a isotype control (Biolegend, 10.9 μg/ml) diluted in RPMI + FCS for 30 min at 37 °C. Afterwards, NK cells were cultured alone or in co-culture with *A*. *fumigatus* germ tubes (MOI 0.5) at a NK cell concentration of 1 × 10^6^ cells/ml. CD56 blocking antibody and isotype control were 4 fold diluted during culture. After 9 h, cells were harvested and CD56 and CD69 expression was evaluated by flow cytometry. Supernatants of co-cultures were stored at −20 °C until a ProcartaPlex^TM^ or ELISA was performed.

### Soluble CD56 protein binding

Different concentrations (0, 1, 5, 10 μg/ml) of soluble CD56 (120 kD, R&D) were incubated with live *A*. *fumigatus* germ tubes (0.5 × 10^6^ cells/ml) on poly-D-lysine coated 8-well Lab-Tek coverglass chambers (Thermo Fisher Scientific) for 6 h at 37 °C. Samples were prepared and stained with mAB CD56 mouse-anti-human Alexa Fluor 647 as described in the section “*Fluorescence microscopy*”. Confocal laser scanning microscopy (CLSM) images of the CD56 -*A*. *fumigatus* interaction were acquired as described in the section “*Fluorescence microscopy*”.

### Cytochalasin D and colchicine treatment

NK cells were treated with 10 μM cytochalasin D (Sigma), 10 μM colchicine or the perspective DMSO and ethanole control for 30 min at 37 °C. NK cells were cultured alone or with *A*. *fumigatus* germ tubes (MOI 0.5) for 0, 3, 6 and 9 h in the presence of 5 μM cytochalasin D or colchicine or the perspective DMSO or ethanol controls. Expression of CD56 was determined using flow cytometry.

### Length and amount of CD56 fluorescent interaction sites

NK cell-*A*. *fumigatus* co-cultures were prepared on Lab-Tek coverglass chambers as described under the section “*Fluorescence microscopy*”. In order to evaluate the volume of the CD56 – *A*. *fumigatus* interaction site, image stacks were recorded from four different time points of co-incubation by CLSM. The number and length of CD56 interaction sites after 3, 6, 9, and 12 h were determined by Fiji and the 3D object counter plugin^54^.

### Scanning electron microscopy (SEM)

NK cells were seeded alone or with *A*. *fumigatus* germ tubes (MOI 0.5) on microscopic cover slips (10 mm, Hartenstein) coated with poly-D-lysine (Sigma Aldrich). 3 h post incubation, samples were washed with PBS and were fixed for 12–18 h at 4 °C in a solution of 2.5% glutaraldehyde (Merck), 2.5 mM MgCl_2_, 50 mM KCl, and 50 mM cacodylic acid, pH 7.2. Afterwards, samples were washed with 50 mM cacodylic acid, pH 7.2 and then, dehydrated stepwise with acetone, critical point dried (critical point dryer: BAL-TEC CPD 030) and metal coated (sputter coater BAL-TEC SCD 005) with gold-palladium. Specimens were examined with a field emission scanning electron microscope (JEOL JSM-7500F) using a detector for secondary electrons (SEI detector) at 5 kV and a magnification of ×10,000.

### Fluorescence microscopy

NK cells were cultured alone or with *A*. *fumigatus* (MOI 0.5) on poly-D-lysine coated 8-well Lab-Tek coverglass chambers for 3, 6, 9 or 12 h. Then, samples were fixed in 0.67% formaldehyde for 30 min. After blocking with 5% BSA in HBSS for 30 min, samples were stained with mAB CD56 mouse-anti-human Alexa Fluor 647 (1:50, Biolegend) and fixed in 2% formaldehyde for 20 min. CLSM images of the NK-*Aspergillus* interaction were acquired with a LSM700 system (Carl Zeiss) with a plan-apochromat 63 ×/1.40 oil immersion objective. *D*STORM imaging of *A*. *fumigatus* incubated NK cells and control NK cells was performed in photoswitching buffer (100 mM mercaptoethylamine in PBS pH 7.4). 2D measurements were conducted on an inverted wide-field fluorescence microscope (IX-71; Olympus) as described previously^20, 55, 56^. For each measurement 15,000 images with an exposure time of 20 ms and irradiation intensity of ~7 kW/cm^2^ were recorded using highly inclined and laminated optical sheet (HILO) illumination.

For 3D *d*STORM measurements^57^ an Axio Observer.Z1 (Carl Zeiss Microscopy) equipped with a water-immersion objective (LD C-Apochromat 63×/1.15 W Corr M27; Carl Zeiss Microscopy) was used. Fluorophores were excited with a 150 mW 640 nm laser (iBeam Smart 640-S; Toptica) which was spectrally cleaned (MaxDiode LD01-640/8; Semrock). Emission and excitation light was separated using a dichroic mirror (BrightLine Di01-R405/466/532/635-25 × 36; Semrock) and a bandpass filter (ZET405/488/532/642 m, Chroma) and the wavelength range of the emitted light was specified further with a single bandpass filter (E700/75 m; Chroma). Astigmatism of the point spread function (PSF) was introduced by a 250 mm achromatic cylindrical lens (Thorlabs). Fluorophores were detected by an EMCCD camera (iXon Ultra DU897U-CSO; Andor). At least 30,000 frames at a frame rate of 54 Hz were collected for each 3D measurement. The open source rapi*d*STORM^58^, version 3.3.1 software was used to reconstruct *d*STORM images from the recorded 2D and 3D image stacks.

### Statistical analysis

A two-way ANOVA or a two-tailed, paired Student’s t-test was used to evaluate statistical significance. *P*-values < 0.05 were considered statistically significant. Statistics were calculated using GraphPad Prism 5 software.

#### Ethics approval

All methods were performed in accordance with the relevant guidelines and regulations. Informed consent has been obtained for study participation and for publication of information, as requested by the Ethics Committee of the University Hospital of Wuerzburg (permit #302/15).

## Electronic supplementary material

Supplementary Video

Supplementary Information
