# Supplementary material for: CD56 Is a Pathogen Recognition Receptor on Human Natural Killer Cells
Source: Sci Rep. 2017 Jul 21;7:6138. doi: 10.1038/s41598-017-06238-4 (PMC5522490; doi:10.1038/s41598-017-06238-4)
Supplement: Supplementary file 2 — Supplementary Information [file 41598_2017_6238_MOESM2_ESM.pdf]

CD56 Is a Pathogen Recognition Receptor on Human Natural Killer Cells

Sabrina Ziegler, Ph. D., Esther Weiss, M.Sc., Anna-Lena Schmitt, Jan Schlegel, Anne Burgert, Ulrich Terpitz, Ph. D., Markus Sauer, Ph. D., Lorenzo Moretta, M.D., Simona Sivori, Ph.D., Ines Leonhardt, Ph. D., Oliver Kurzai, M.D., Hermann Einsele, M.D., Juergen Loeffler, Ph.D.

Supplementary Figure 1:

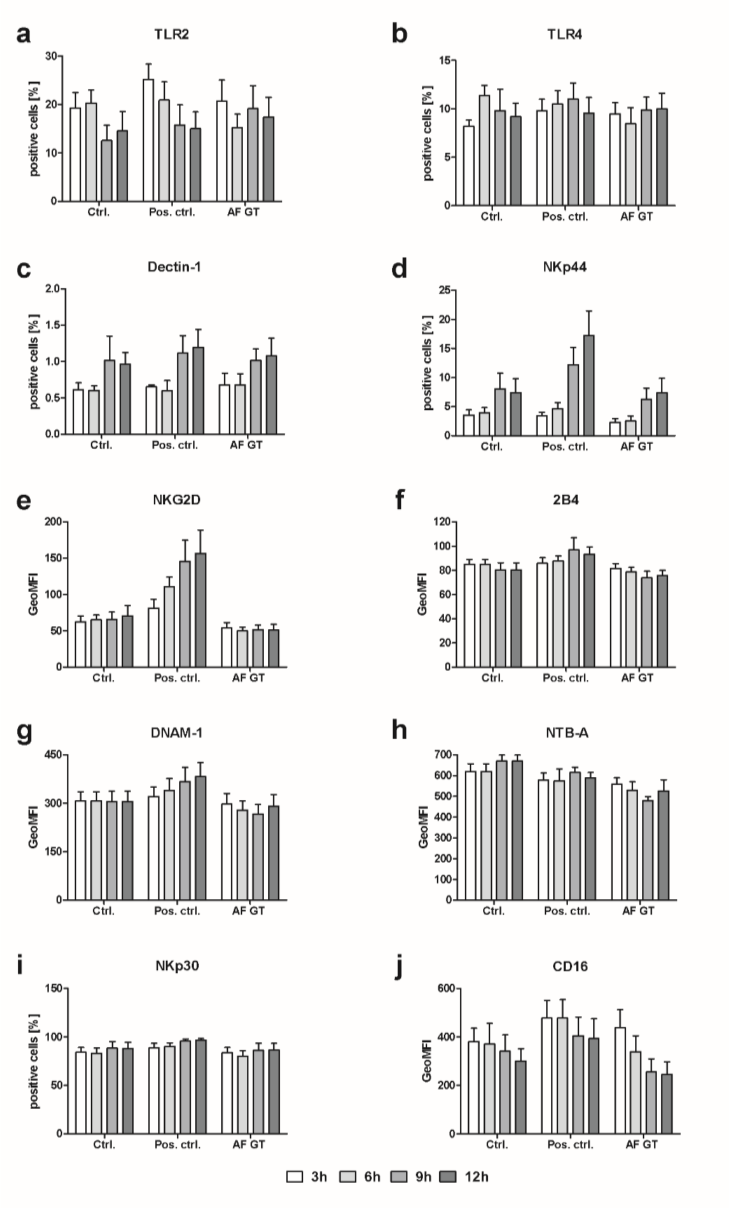

Expression of PRRs and NK cell activating receptors is not altered by the presence of A. fumigatus. Primary NK cells were pre-treated overnight with IL-2 (1000 U/ml). NK cells were treated with 500 U/ml IL-15 and IL-2 (Pos. ctrl.), with *A. fumigatus* germ tubes (AF GT) at MOI 0.5 or left untreated (Ctrl.) for 3 h, 6 h, 9 h and 12 h. Flow cytometry was performed to analyse a) TLR-2, b) TLR-4, c) Dectin-1, d) NKp44, e) NKG2D, f) 2B4, g) DNAM-1, h) NTB-A, i) NKp30 and j) CD16 cell surface expression. NK cells were defined as $NKp46^{+}CD3^{-}$. For TLR-2, TLR-4, Dectin-1, NKp30 and NKp44 percent positive cells are shown whereas for NKG2D, 2B4, DNAM-1, CD16 and NTB-A the geometric mean fluorescence is visualized. Data are represented as mean + SEM of a) n = 5, b) n = 6, c) n = 4, d) n = 4, e) n= 4, f) n = 3, g) n = 3, h) n = 3, i) n = 5 and j) n = 5 independent experiments.

Supplementary Figure 2:

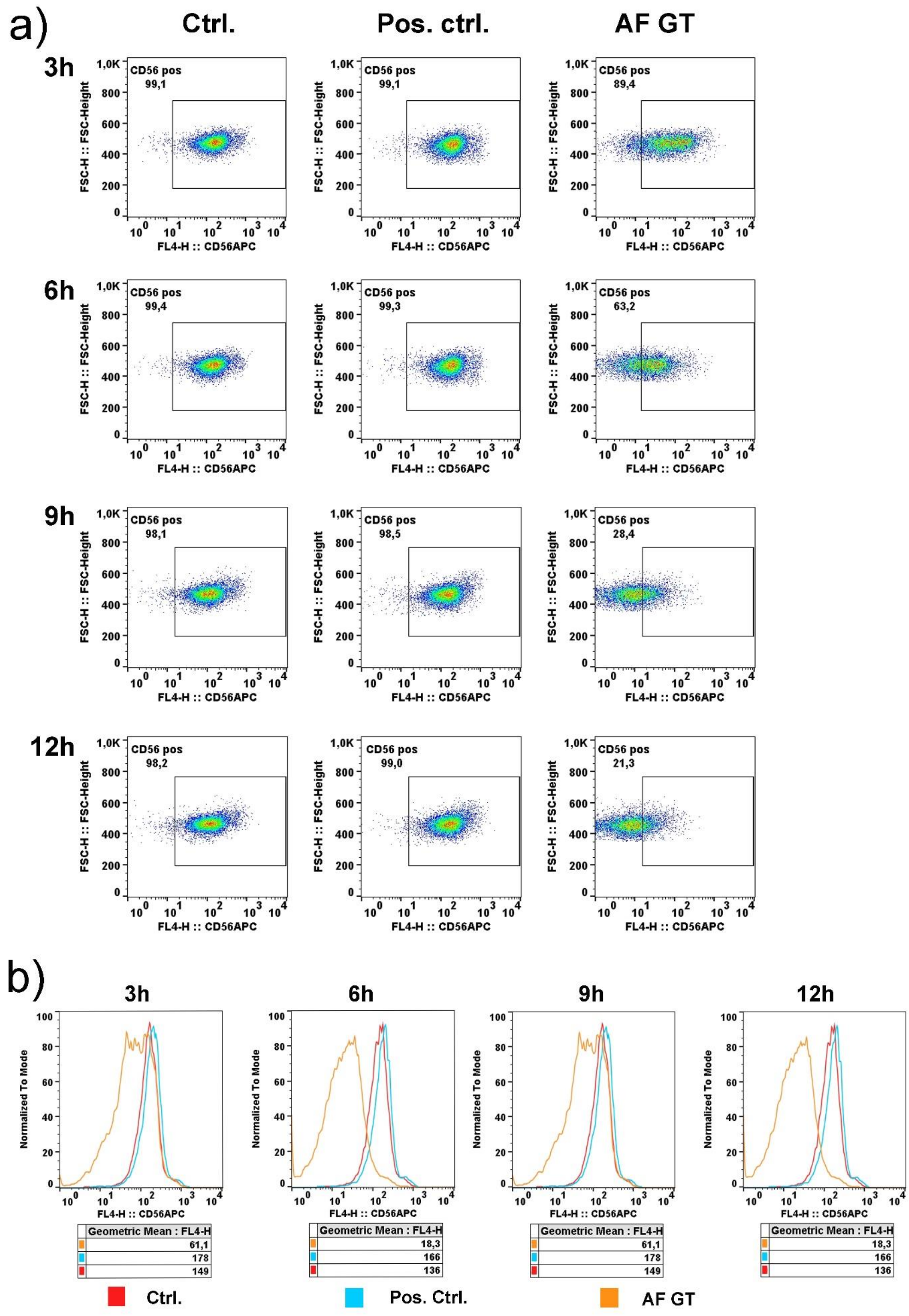

Percent positive cells and geometric mean fluorescence of CD56 was significantly reduced after treatment with A. fumigatus. Primary NK cells were pre-treated overnight with IL-2 (1000 U/ml). NK cells were treated with 500 U/ml IL-15 and IL-2 (Pos. ctrl.), with *A. fumigatus* germ tubes (AF GT) at MOI 0.5 or left untreated (Ctrl.) for 3 h, 6 h, 9 h and 12 h. Flow cytometry was performed to analyse a) percent positive CD56 cells and b) the geometric mean fluorescence of CD56 NK cells. NK cells were defined as $NKp46^{+}CD3^{-}$. Data are representative of 4 independent experiments.

Supplementary Figure 3:

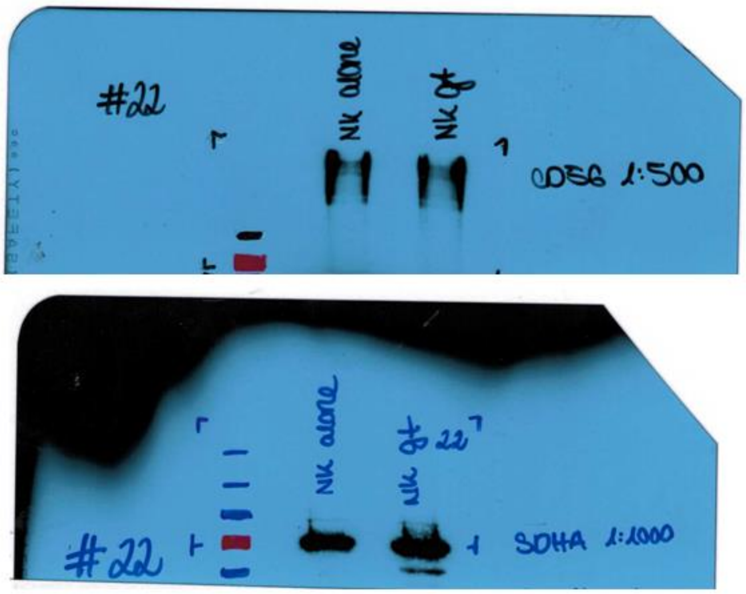

CD56 western blot analysis. Protein concentrations were visualized by western blot analyses 4 h after co-cultivation. Developing time of films took 20-60 sec. Representative data of five independent experiments.

Supplementary Figure 4:

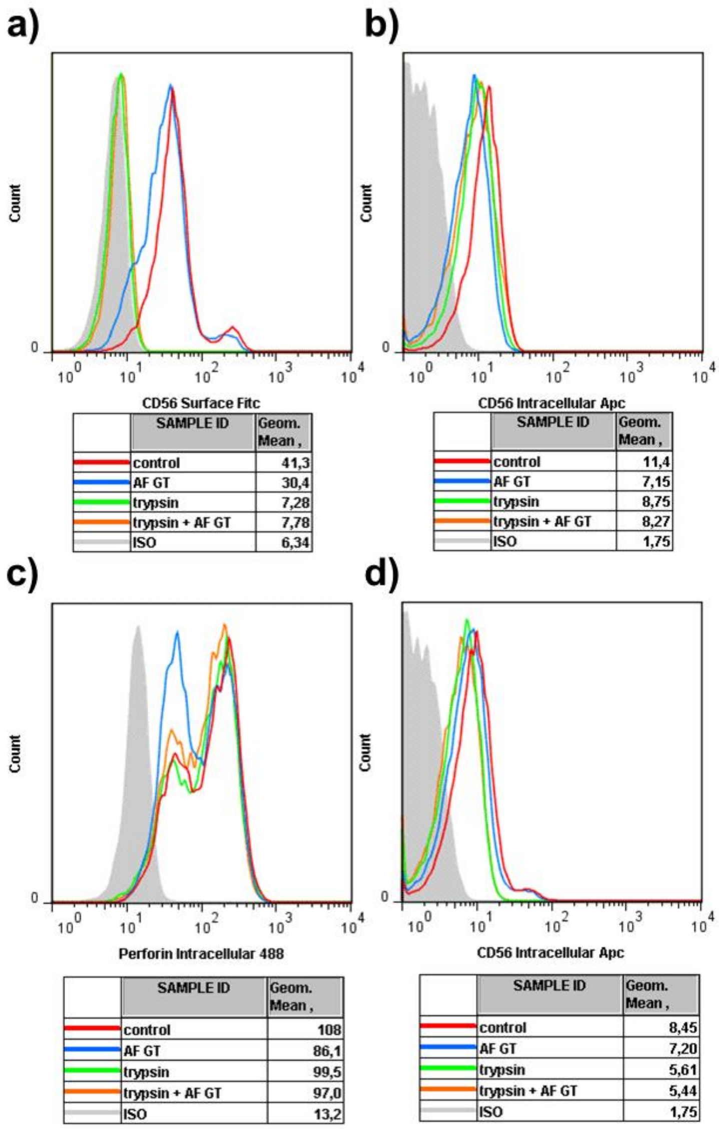

CD56 reduction in the percentage of positive cells on the surface is not induced by internalization. NK cells were cultured alone or co-cultured with *A. fumigatus* germ tubes (MOI 0.5) for 6 h. NK cells were stained for extracellular (a) and intracellular (b, d) levels of CD56. To detect only intracellular CD56 signal, NK cells were treated with trypsin prior to intracellular staining (a, b, d). To provide a further positive control for intracellular staining, cells were stained for intracellular perforin and CD56 in the same flow cytometric sample (c, d). Red line: NK cells (control); blue line: NK cells treated with *A. fumigatus* (AF GT); green line: NK cells treated with trypsin (trypsin); orange line: NK cells treated with trypsin and *A. fumigatus* (trypsin + AF GT). e) Protein concentrations were visualized by western blot analyses 4 h after co-cultivation. Blots were cropped and image processing was performed by Adobe Photoshop software. Representative data of three independent experiments. Data of a), b), and c) are represented as mean +SEM for a) n = , b) n = and c) n = 3). Significant differences are marked with an asterisk (*** $p < 0.005$).

Supplementary Figure 5:

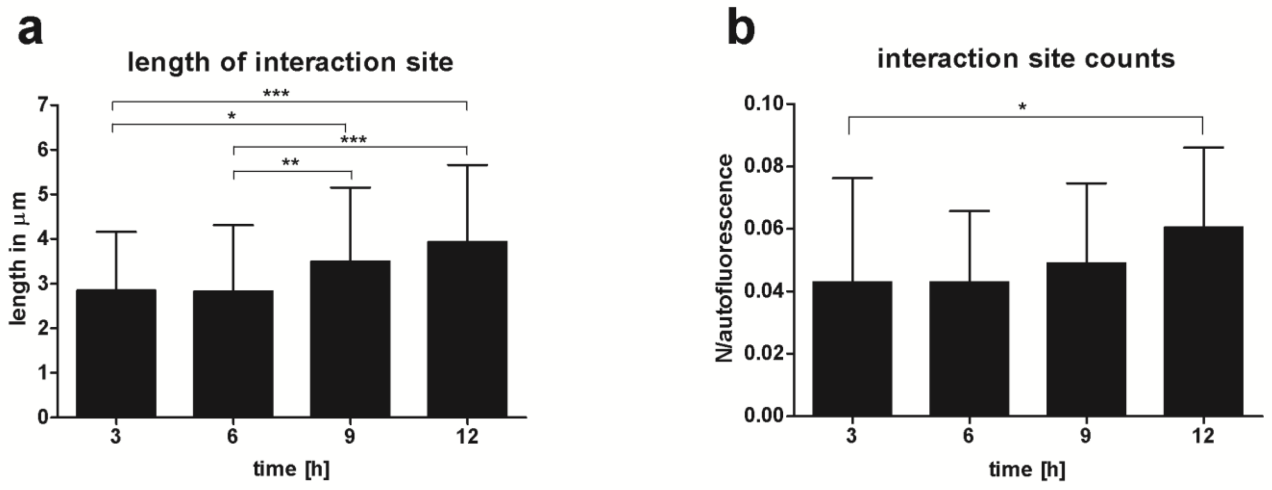

CD56 interaction site is increasing with time. NK cells were co-cultured with *A. fumigatus* germ tubes (MOI 0.5) on poly-D-lysine coated 8-well Lab-Tek coverglass chambers for 3, 6, 9, and 12 h. Samples were fixed and stained with anti-CD56 Alexa Fluor 647 antibody and were analysed by CLSM. a) The lengths of 35 interaction sites per time point were measured by FiJi software. B) The number of interaction sites was measured from 10 picture stacks per time point by the 3D object counter plugin from FiJi software. The number of interaction sites was normalized to the measured fungal autofluorescence. Data are represented as mean + SD of a), b) n = 3 independent experiments. Significant differences are indicated by asterisk (* $p < 0.05$, ** $p < 0.01$, *** $p < 0.005$) analysed by Kruskal-Wallis test.

Supplementary Figure 6:

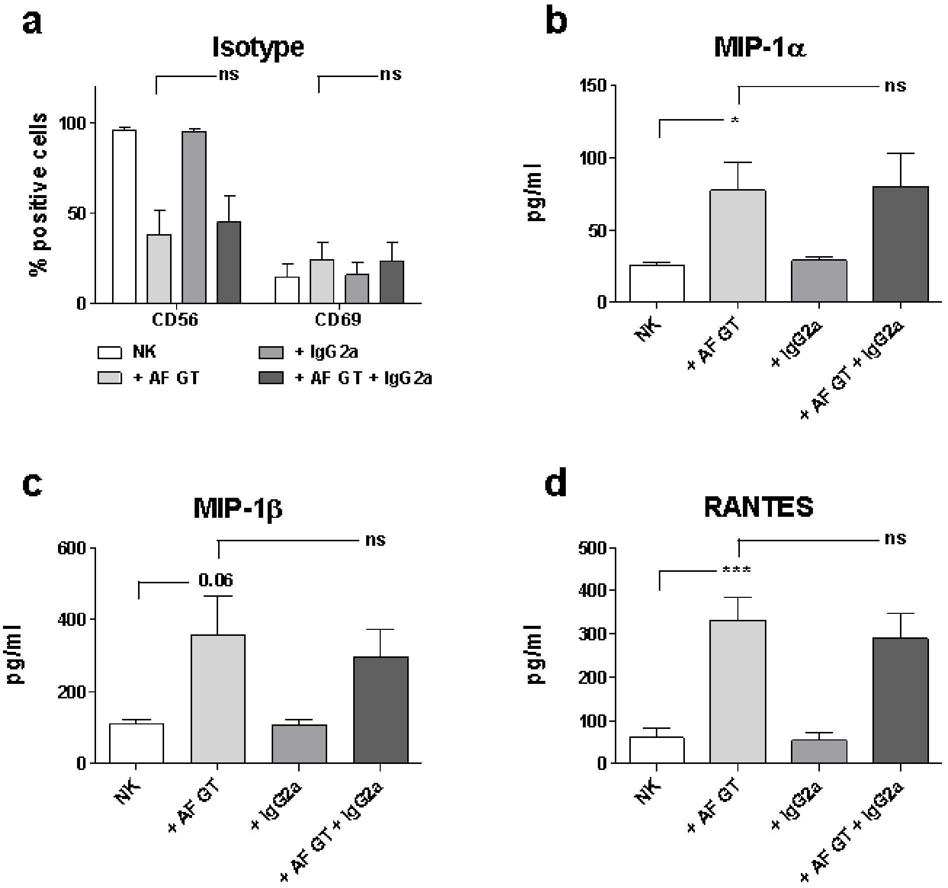

Treatment with mouse IgG2a antibody does not influence CD56 or CD69 expression nor the secretion of CCL 3, 4, and 5. NK cells were treated with 10.9 µg/ml mouse IgG2a antibody (Biolegend) diluted in RPMI + FCS in a concentration of 4 x 10^6 cells/ml for 30 min at 37 °C. NK cells were cultured alone or in co-culture with *A. fumigatus* germ tubes (MOI 0.5) for 9 h while the antibody was 4-fold diluted to 2.7 µg/ml. NK cells were analysed by the percentage of CD56 and CD69 (a) positive cells and the secretion (in pg/ml) of MIP-1α, (b) MIP-1β (c) and RANTES (d). Data are represented as mean ± SEM of four independent experiments. To analyse the influence of the IgG2a isotype control on fungal treated NK cells, + AF GT was compared to + AF GT + IgG2a with a student's t-test.

Video 1:

Movie of 3D stack showing the interaction site of a NK cell and *A. fumigatus*. NK cells were incubated for 3 h with *A. fumigatus* (MOI 0.5) on poly-D-lysine coated 8-well Lab-Tek coverglass chambers. Thereafter samples were fixed and stained with anti CD56-Alexa Fluor 647. Co-cultures were visualized by 3D *d*STORM.
